# Current status and future perspective of postoperative treatment for locally advanced squamous cell carcinoma of the head and neck

**DOI:** 10.1093/jjco/hyae029

**Published:** 2024-03-07

**Authors:** Naomi Kiyota, Makoto Tahara, Akihiro Homma

**Affiliations:** Department of Medical Oncology/Hematology, Kobe University Hospital, Kobe, Japan; Kobe University Hospital Cancer Center, Kobe, Japan; Department of Head and Neck Medical Oncology, National Cancer Center Hospital East, Kashiwa, Japan; Department of Otolaryngology-Head and Neck Surgery, Faculty of Medicine and Graduate School of Medicine, Hokkaido University, Sapporo, Japan

**Keywords:** head and neck cancer, adjuvant, radiotherapy, cisplatin, immunotherapy

## Abstract

Surgery remains a foundation of treatment for locally advanced squamous cell carcinoma of the head and neck. For postoperative patients at high risk of recurrence, however, surgery by itself is not enough, and improvement in survival requires postoperative treatment. Unlike the case with most other malignancies, the standard postoperative treatment for locally advanced squamous cell carcinoma of the head and neck patients with high-risk factors for recurrence is radiotherapy or chemoradiotherapy with cisplatin. However, chemoradiotherapy with cisplatin at a dose of 100 mg/m^2^ once every 3 weeks has raised discussion over insufficient cisplatin delivery due to high-dose-related toxicity. As a possible solution, a recent randomized trial of the JCOG1008 has proved the non-inferiority of postoperative chemoradiotherapy with weekly cisplatin at a dose of 40 mg/m^2^ to 3-weekly cisplatin in terms of overall survival. Here, this review article focuses on current evidence and future perspectives of postoperative treatment for locally advanced squamous cell carcinoma of the head and neck.

## Teaser

The JCOG1008 trial established a new standard treatment for head and neck cancer at high risk for recurrence, demonstrating the non-inferiority of postoperative chemoradiotherapy with weekly cisplatin to 3-weekly cisplatin.

## Introduction

Global cancer incidence and mortality data report an annual incidence of head and neck cancer of around 870 000 in 2020. Around 440 000 patients died from the disease, accounting for 4.5% of cancer deaths (1). Approximately 50% of head and neck cancer patients present with Stage III/IV, and the prognosis of these patients is generally unfavorable. Patients with locally advanced squamous cell carcinoma of the head and neck (SCCHN) are commonly treated with surgery as primary intervention. In cases with risk factors for recurrence based on surgical pathology, standard postoperative treatment involves radiotherapy (RT) or chemoradiotherapy (CRT) (2–5).

Of particular note, for postoperative patients at high risk of recurrence—namely a microscopically positive margin and/or extra nodular extension (ENE)—CRT with cisplatin at a dose of 100 mg/m^2^ has been the standard of care (2–4). Rates of local relapse and distant metastasis relapse after postoperative CRT (POCRT) are high, however, at up to 20%, and 5-year survival rate is around 50% (2,3,6). Furthermore, only around 60% of patients previously reported in the United States and Europe completed three cycles of high-dose cisplatin once every 3 weeks and meet the criteria for dose reduction (2,3). Moreover, rates of severe acute and late adverse reactions remain high, and include renal impairment, hearing disturbance, myelosuppression and infections such as aspiration pneumonia. The unmet need in POCRT for SCCHN patients with high-risk factors for recurrence includes not only greater efficacy but also lower toxicity.

In this review article, we focus on current evidence and future perspectives of postoperative treatment for SCCHN patients with high-risk factors for recurrence.

## Standard treatment for SCCHN patients with high-risk factors for recurrence

As noted in the Introduction, although surgery is a mainstay of treatment for locally advanced SCCHN, prognosis in patients with high-risk factors for recurrence is poor without proper postoperative treatment. For these patients, many previous randomized clinical trials have reported that POCRT is beneficial (2,3,7–9); the most pivotal among them is RTOG95-01 and EORTC22931.

The RTOG95-01 trial was a Phase III randomized control trial for postoperative SCCHN patients with any of the risk factors for recurrence listed in Table 1, namely multiple cervical lymph node metastases (≥2), microscopic resection margin positivity and ENE. Risk factors for recurrence in RTOG95-01 were based on a combined analysis of data from the RTOG 85-03 and RTOG 88-24 trials (10). In RTOG95-01, POCRT with 3-weekly cisplatin at a dose of 100 mg/m^2^ provided a significantly superior locoregional control (LRC) rate and disease-free survival (DFS) but no superiority in overall survival (OS) (2). The EORTC22931 trial was a Phase III randomized control trial for postoperative SCCHN patients with any of the risk factors for recurrence listed in Table 1; in addition to microscopic resection margin positivity and ENE, these included Stage III/IV disease, level 4/5 lymph node metastasis in oropharyngeal cancer/oral cavity cancer, perineural infiltration, as well as signs of vascular tumor embolism (3,4,11,12). In EORTC22931, POCRT with 3-weekly cisplatin at a dose of 100 mg/m^2^ provided significantly superior LRC rate, DFS and OS compared with PORT (3). Allowing that these two trials differed in some of the definitions of risk factors for recurrence, their combined analysis nevertheless showed that POCRT with 3-weekly cisplatin for patients with risk factors for recurrence improved OS with a hazard ratio (HR) of 0.776. This improvement was particularly noted among patients with risk factors common to the two trials (microscopic margin positivity and ENE), with a HR of 0.702. In stark contrast, however, patients without these common risk factors showed no survival benefit of POCRT with 3-weekly cisplatin (4). Consequently, POCRT with 3-weekly cisplatin has become the standard treatment for postoperative SCCHN patients with high-risk factors for recurrence. The feasibility of POCRT with 3-weekly cisplatin at a dose of 100 mg/m^2^ has also been investigated outside the U.S. and Europe: in a Phase II study in Japan, 80% (20/25) of patients were able to complete protocol-defined treatment (13). Moreover, the safety profile of this study was similar to that of previous studies of POCRT with cisplatin at a dose of 100 mg/m^2^ (2,3). Thus, POCRT with cisplatin at a dose of 100 mg/m^2^ appeared to be feasible in non-western countries, and has accordingly been recognized as the global standard treatment for these patients.

**Table 1:** Differences in risk factors for recurrence between RTOG95-01 and EORTC22931

**Risk factors only in RTOG** **(intermediate risk for recurrence)**	**Risk factors common to RTOG and EORTC** **(high risk for recurrence)**	**Risk factors only in EORTC** **(intermediate risk for recurrence)**
Multiple cervical LN metastasis (≥ 2)	Microscopic resection margin positivity, ENE	Stage III/IV disease, perineural infiltration, level 4/5 LN metastasis in oropharyngeal cancer/oral cavity cancer, vascular tumor embolism

LN, lymph node; ENE, extranodular extension

## Another potential option for the SCCHN patients with high-risk factors for recurrence

### Postoperative radiotherapy

Prognosis of Stage III/IV resectable locally advanced SCCHN remains poor, and PORT after surgery remains a treatment option for this cancer (14). PORT for patients with resectable locally advanced SCCHN commonly involves a total dose of 60 to 66 Gy, with conventional fractionation at 1.8 to 2.0 Gy per day (2,3). Five-year survival rate remains as low as 40% (15), however, and PORT is now indicated for patients with risk factors other than high risk for recurrence (intermediate risk factors for recurrence), and also for those with high-risk factors for recurrence who are unsuitable for POCRT with high-dose cisplatin due to insufficient organ function (renal impairment, hearing impairment, etc.).

### Postoperative CRT with chemotherapeutic agents other than cisplatin

Given that carboplatin is frequently used clinically as an alternative to cisplatin, POCRT with carboplatin may be a potential treatment option for SCCHN patients with high-risk factors for recurrence. Argiris et al. reported a Phase III study, which compared PORT and POCRT with carboplatin for SCCHN patients with high-risk factors for recurrence (16). PORCT with carboplatin failed to show superiority to PORT in terms of DFS and OS, however, and is therefore not recommended for these patients.

As another approach to avoiding the toxicity of high-dose cisplatin, cetuximab plus weekly cisplatin at a dose of 30 mg/m^2^, and cetuximab plus weekly docetaxel at a dose of 15 mg/m^2^ were investigated as potentially promising chemotherapeutic regimens for POCRT in a randomized Phase II trial of RTOG0234 (17). In that trial, the two regimens were compared with historical data from RTOG95-01 of POCRT with cisplatin at a dose of 100 mg/m^2^ (2). Results showed that POCRT with cetuximab plus weekly cisplatin exhibited a non-significant improvement in DFS with a HR of 0.76 [95% confidence interval (CI), 0.54–1.06], while POCRT with cetuximab plus weekly docetaxel exhibited a significant improvement in DFS with a HR of 0.69 (95%CI, 0.50–0.96). From these results, POCRT with cetuximab plus weekly docetaxel is now under investigation as a challenge arm of an ongoing Phase II/III trial of the RTOG1216 trial, which will be described later. POCRT with cetuximab plus weekly docetaxel is therefore one candidate for a potential alterative regimen to high-dose cisplatin, and the results of the ongoing RTOG1216 trial (NCT01810913) are awaited.

### Postoperative chemotherapy

In contrast to other cancer types, the role of postoperative chemotherapy for SCCHN patients remains unclear. Although POCRT has been extensively investigated, comparative data from randomized trials with or without postoperative chemotherapy for SCCHN patients are scarce. (15, 18–20). Among the data that are available, however, these randomized trials failed to show the efficacy of postoperative chemotherapy in this setting. A randomized study (*N* = 180) reported from Japan compared an oral fluoropyrimidine (S-1) with tegafur/uracil (UFT) for SCCHN patients treated with curative treatment; although S-1 showed significantly improved OS compared with UFT [HR, 0.46 95% CI, 0.22–0.93], UFT alone was not used as the established control arm (21), and the efficacy of postoperative adjuvant chemotherapy after curative treatment which includes surgical resection for SCCHN patients remains to be determined.

## Optimal dosage of cisplatin, new evidence from randomized controlled trials

As mentioned above, 3-weekly cisplatin at dose of 100 mg/m^2^ is the best-regarded standard treatment for postoperative LA-SCCHN patients with high-risk factors for recurrence. Nevertheless, the 3-weekly dosage did raise discussion about insufficient delivery due to high-dose-related toxicities (2,3). In contrast, weekly cisplatin has been used as a possible treatment option with a better safety profile. This followed meta-analyses which compared 3-weekly and weekly cisplatin, which suggested that the two approaches have efficacy, albeit that some results were conflicting (22–34). In addition to cisplatin dosage, the cumulative dose of cisplatin during RT for locally advanced SCCHN significantly correlates with survival and must also be considered. A cumulative dose of 200 mg/m^2^ during RT is suggested to be sufficient to achieve an additive effect with RT irrespective of cisplatin dosage (35,36). Regarding POCRT, two important randomized controlled trials (RCTs) of 3-weekly vs. weekly cisplatin have appeared, one by Tata Memorial Hospital (TMH) in India and the second by the Japan Clinical Oncology Group (JCOG) (30,37). As summarized in Table 2, the TMH trial found that weekly cisplatin at a dose of 30 mg/m^2^ was inferior to 3-weekly CDDP at a dose of 100 mg/m^2^ with regard to LRC rate. On the contrary, the JCOG1008 trial reported that weekly cisplatin at a dose of 40 mg/m^2^ was non-inferior to 3-weekly cisplatin at a dose of 100 mg/m^2^ using OS as the primary endpoint. Moreover, with regard to toxicity, the two studies both reported that gastrointestinal and hematological toxicities, infection, hearing impairment and renal impairment were generally better with weekly administration. It was concluded that weekly cisplatin at a dose of 40 mg/m^2^ concurrent with RT is acceptable as a treatment option for postoperative patients with LA-SCCHN at high risk of recurrence.

**Table 2:** Comparison of the TMH and JCOG1008 trials

**Trial**	**TMH trial**	**JCOG1008**
**Phase**	III	II/III
**Trial design**	Non-inferiority design	Non-inferiority design
**Eligible patients**	Locally advanced SCCHN 7% Postoperative high-risk SCCHN 93%	- Postoperative high-risk SCCHN
**Number of patients**	300	261
**Control arm**	3-weekly cisplatin 100 mg/m^2^	3-weekly cisplatin 100 mg/m^2^
**Test arm**	Weekly cisplatin 30 mg/m^2^	Weekly cisplatin 40 mg/m^2^
**Radiation therapy**	2D or 3D CRT or IMRT	3D CRT or IMRT (63%)
**Total dose**	Definitive, 70 Gy/35 Fr, Postoperative, 66 Gy/33Fr	Postoperative, 66 Gy/33Fr
**Primary endpoint**	Locoregional control (LRC)	Overall survival (OS)
3-weekly vs. weekly	2-year LRC 73.1% vs. 58.5% HR 1.76 (95% CI 1.11–2.79), *P* = 0.014	3-year OS 59.1% vs. 71.6% HR 0.69 (99.1%CI: 0.37–1.27), *P* for non-inferiority = 0.0027
**Secondary endpoint**	Overall survival (OS)	Relapse-free survival (RFS)
3-weekly vs. weekly	Median OS NR vs. 39.5 months HR 1.14 (95%CI 0.79–1.65), *P* = 0.48	3-year RFS 53.0% vs. 64.5% HR 0.71 (95%CI 0.48–1.06)
**Toxicities**	Better in weekly arm	Better in weekly arm
**Median cumulative CDDP**		
3-weekly vs. weekly	300 mg/m^2^ vs. 210 mg/m^2^	280 mg/m^2^ vs. 239 mg/m^2^

SCCHN, squamous cell carcinoma of the head and neck; NR, not reached; Gy, gray; Fr, fraction; 3D CRT, three-dimensional conformal radiation therapy; IMRT, intensity-modulated radiation therapy

For weekly cisplatin in the definitive setting, Sharma et al. reported at ASCO 2022 a Phase III randomized trial (ConCERT trial, *N* = 278) of CRT with 3-weekly cisplatin (100 mg/m^2^) vs. weekly cisplatin (40 mg/m^2^) in patients with LA-SCCHN (38). CRT with weekly cisplatin was shown to be non-inferior to 3-weekly cisplatin using LRC rate as primary endpoint. We await peer-reviewed publication of these results. Furthermore, NRG-HN009 (NCT05050162) is a Phase II/III RCT of 3-weekly vs. weekly administration now underway in a larger sample size for locally advanced SCCHN (*N* = 1250). A weekly regimen in the definitive setting as standard clinical practice for locally advanced SCCHN should not be adopted until the results of these studies are reported.

## Postoperative treatment for the patients with p16-positive oropharyngeal cancer

Following reports that prognosis in oropharyngeal cancer is more favorable among those patients who are human papilloma virus (HPV)-positive (39,40), treatment deintensification for HPV-related oropharyngeal cancer is now under extensive investigation. Against this background, the randomized Phase II E3311 trial was conducted for HPV-positive oropharyngeal cancer patients treated with transoral surgery (TOS). The study enrolled HPV-positive oropharyngeal cancer patients with cT1-2/N1-2b (UICC 7th edition) who were treated with TOS. Patients with no risk factors for recurrence were allocated to an observation cohort (Arm A), while those with high-risk factors for recurrence under this trial (microscopic margin positivity, > 1 mm ENE, 5 or more lymph node metastases) were allocated to POCRT 66 Gy/33 fractions with weekly cisplatin at a dose of 40 mg/m^2^ (Arm D). Patients with intermediate risk factors for recurrence—namely margin <3 mm, ENE within 1 mm and 2–4 lymph node metastases—were randomly allocated to reduced PORT with 50 Gy (Arm B) or standard PORT with 60 Gy (Arm C). Results were promising: the primary endpoint of 2-year progression-free survival was 96.9% (90%CI, 91.9–100) in Arm A, 94.9% (90%CI, 91.3–98.6) in Arm B, 96.0% (90%CI, 92.8–99.3) in Arm C and 90.7% (90%CI, 86.2–95.4) in Arm D. These results are particularly impressive for patients with intermediate risk who were treated with reduced dose PORT and provide sound justification for a Phase III trial. In part confirmation of treatment deintensification for HPV-positive oropharyngeal cancer, a large-scale retrospective analysis of over 14 000 patients from the National Cancer Data Base reported that the addition of chemotherapy to PORT was not associated with an improvement in treatment outcomes for p16-positive oropharyngeal cancer patients with high-risk factors for recurrence (41). Nevertheless, any implementation of deintensification strategies in clinical practice should be approached with caution, since many randomized trials of deintensification for HPV-positive oropharyngeal cancer patients failed to show the non-inferiority of deintensification to the standard treatment strategy (42–44).

## Future perspectives for adjuvant treatment for postoperative HNSCC

The current standard treatment for postoperative locally advanced SCCHN patients with high-risk factors for recurrence is adjuvant CRT with cisplatin. Five-year OS in this setting remains low, however, at around 50–70%, (2,3,37) while even with a weekly cisplatin regimen—expected to be less toxic—around 80% of patients experience at least one Grade 3 or higher adverse event. These findings highlight a clear need for more effective and less toxic POCRT (37).

Table 3 summarizes ongoing Phase III investigations of more effective postoperative treatment for LA-SCCHN patients. IHN-01 (NCT00957086) is investigating the addition of nimotuzumab, an anti-EGFR antibody, to POCRT with 3-weekly cisplatin. The final result of IHN-01 is anticipated. This anticipation is despite the fact that the addition of lapatinib, a tyrosine kinase inhibitor which targets both EGFR and HER2, to POCRT with 3-weekly cisplatin failed to show an improvement in DFS (HR 1.10, 95% CI: 0.85–1.43) or OS (HR 0.96, 95% CI: 0.73–1.25). X-RAY VISION is a randomized trial in postoperative high-risk SCCHN patients who are unfit for high-dose cisplatin, in comparison with PORT with xevinapant or placebo. Xevinapant is an IAP (inhibitor of apoptosis protein) inhibitor, which demonstrated promising efficacy in combination CRT with high-dose cisplatin for locally advanced SCCHN patients (45). All of the other ongoing trials are investigating the addition of anti-PD-1/PD-L1 antibodies to postoperative treatment strategies. Among these, RTOG1216, a randomized phase II/III trial for postoperative high-risk LA-SCCHN patients, is comparing POCRT with weekly cisplatin with either cetuximab plus weekly docetaxel or with atezolizumab (anti-PD-L1 antibody) plus weekly cisplatin. This trial is investigating the superiority of POCRT with cetuximab plus weekly docetaxel, subsequent to a randomized phase II trial of RTOG0234, as well as POCRT with atezolizumab plus weekly cisplatin to POCRT with weekly cisplatin (17). Since the anti-PD-1 antibodies nivolumab and pembrolizumab show a survival benefit in recurrent or metastatic SCCHN, this approach may be promising (46,47). For the locally advanced setting, however, the addition of the anti-PD-L1 antibody avelumab or anti-PD-1 antibody pembrolizumab to CRT also failed to improve treatment outcomes in respective randomized trials (JAVELIN Head and Neck 100, KEYNOTE-412) (48,49). Overall, these results indicate the need to carefully await the results of ongoing Phase III trials of the integration of immune checkpoint inhibitors into postoperative treatment strategies.

**Table 3:** Ongoing Phase III trials for postoperative settings

**Trial** **(NCT No.)**	**Design**	***N***	**Patients**	**Experimental arm**	**Control arm**	**Primary endpoint**
IHN-01 (00957086)	PIII, DB	710	Postoperative Int-high risk	3-weekly cisplatin+RT Plus nimotuzumab	3-weekly cisplatin +RT Plus PBO	DFS
X-RAY VISION (05386550)	PIII DB	700	Postoperative High-risk Unsuitable for high-dose cisplatin	Xevinapant+RT	PBO + RT	DFS
IMvoke010 (03452137)	PIII, DB	406	LA-SCCHN After curative Tx	Atezolizumab	PBO	EFS, OS
KN-689 (03765918)	PIII, OL	704	Resectable LA-SCCHN	Pembrolizumab before surgery Surgery High-risk: 3-weekly cisplatin +RT +pembrolizumab Low-risk: RT + pembrolizumab	- Surgery High-risk: 3-weekly cisplatin +RT Low-risk: RT	MPR, EFS
GORTEC 2018-01 (03576417)	PIII, OL	680	Postoperative Int-high risk	Nivolumab (3 weeks before CRT) 3-weekly cisplatin +RT Maintenance nivolumab	3-weekly cisplatin +RT	DFS
RTOG1216 (01810913)	PII/III, OL	613	Postoperative high-risk	Weekly DTX + cetuximab+RT or Weekly cisplatin+atezolizumab+RT	Weekly cisplatin +RT	OS

PIII, Phase III; PII/III, Phase II/III; DB, double blind; OL, open label; LA-SCCHN, locally advanced squamous cell carcinoma of the head and neck; RT, radiotherapy; CRT, chemoradiotherapy; DFS, disease-free survival; EFS, event-free survival; OS, overall survival; MPR, major pathological response

## Conclusions

The standard postoperative treatment for LA-SCCHN patients with high-risk factors for recurrence is POCRT with cisplatin, especially with weekly cisplatin at a dose of 40 mg/m^2^, based on the report of the JCOG1008 trial. Nevertheless, treatment outcomes with POCRT with cisplatin remain unsatisfactory. Hence, greater novelty in the investigational approach to efficacious postoperative treatment is warranted.

## Acknowledgement

We thank Guy Harris DO of Dmed (www.dmed.co.jp) for editing a draft of this manuscript.

## Conflict of interest statement

Dr Kiyota reports grants from Ono Pharmaceutical, Bristol-Meyers Squibb, Astra Zeneca Co., Ltd, Chugai Pharmaceutical Co., Ltd, Boehringer-Ingelheim, Bayer, GSK and Adlai Nortye outside the submitted work; and honoraria from Bayer, Ono Pharmaceutical Co., Ltd, Bristol-Meyers Squibb, Merck Biopharma, Astra-Zeneca Co., Ltd and Merck Sharp & Dohme.

Dr Tahara reports grants and personal fees from Ono Pharmaceutical; grants and personal fees from Bayer; and personal fees from MSD, BMS, Merck Biopharma, Pfizer, Rakuten Medical, Lilly, Boehringer Ingelheim, Eisai, Chugai Pharmaceutical, Daiichi-Sankyo, Janssen Pharmaceutical, Genmab, Astra Zeneca, Abbvie and Astellas outside the submitted work.

Dr Homma reports grants from Japan AMED and the National Cancer Center Research and Development Fund; grants and personal fees from Taiho Pharmaceutical Co., Ltd, ONO Pharmaceutical Co., Ltd, KYORIN Pharmaceutical Co., Ltd, Eisai and Mitsubishi Tanabe Pharma; grants from Otsuka Pharmaceutical Factory, Iwasakidenshi Co., Ltd and Torii Pharmaceutical Co., Ltd; personal fees from Bristol-Myers Squibb K.K., Merck, Bayer Yakuhin, Eli Lilly Japan, Sanofi, Rakuten Medical, Meiji Pharma, Demant Japan K.K., and MSD K.K. outside the submitted work.

## Funding

This work is partially supported by the National Cancer Center Research and Development Funds (2023-J-3) and by AMED under Grant No. 23ck0106842h0001.

## Author contributions

Made substantial contributions to conception, drafting and reviewing the manuscript: N.K.

Made contributions to drafting and reviewing the manuscript: M.T. and A.H.
